# N‐acetylcysteine prevents oxidized low‐density lipoprotein‐induced reduction of MG53 and enhances MG53 protective effect on bone marrow stem cells

**DOI:** 10.1111/jcmm.14798

**Published:** 2019-11-19

**Authors:** Xin Li, Meng Jiang, Tao Tan, Chandrakala A. Narasimhulu, Yuan Xiao, Hong Hao, Yuqi Cui, Jia Zhang, Lingjuan Liu, Chunlin Yang, Yixi Li, Jianjie Ma, Catherine M. Verfaillie, Sampath Parthasarathy, Hua Zhu, Zhenguo Liu

**Affiliations:** ^1^ Department of Endocrinology The First Affiliated Hospital Dalian Medical University Dalian China; ^2^ Center for Precision Medicine and Division of Cardiovascular Medicine University of Missouri School of Medicine Columbia Missouri USA; ^3^ Davis Heart and Lung Research Institute The Ohio State University Wexner Medical Center Columbus OH USA; ^4^ Burnett School of Biomedical Sciences College of Medicine University of Central Florida Orlando FL USA; ^5^ Stem Cell Institute University of Leuven Leuven Belgium

**Keywords:** bone marrow stem cell, membrane damage, MG53, multipotent adult progenitor cells, N‐acetylcysteine, oxidized low‐density lipoprotein

## Abstract

MG53 is an important membrane repair protein and partially protects bone marrow multipotent adult progenitor cells (MAPCs) against oxidized low‐density lipoprotein (ox‐LDL). The present study was to test the hypothesis that the limited protective effect of MG53 on MAPCs was due to ox‐LDL‐induced reduction of MG53. MAPCs were cultured with and without ox‐LDL (0‐20 μg/mL) for up to 48 hours with or without MG53 and antioxidant N‐acetylcysteine (NAC). Serum MG53 level was measured in ox‐LDL‐treated mice with or without NAC treatment. Ox‐LDL induced significant membrane damage and substantially impaired MAPC survival with selective inhibition of Akt phosphorylation. NAC treatment effectively prevented ox‐LDL‐induced reduction of Akt phosphorylation without protecting MAPCs against ox‐LDL. While having no effect on Akt phosphorylation, MG53 significantly decreased ox‐LDL‐induced membrane damage and partially improved the survival, proliferation and apoptosis of MAPCs in vitro. Ox‐LDL significantly decreased MG53 level in vitro and serum MG53 level in vivo without changing MG53 clearance. NAC treatment prevented ox‐LDL‐induced MG53 reduction both in vitro and in vivo. Combined NAC and MG53 treatment significantly improved MAPC survival against ox‐LDL. These data suggested that NAC enhanced the protective effect of MG53 on MAPCs against ox‐LDL through preventing ox‐LDL‐induced reduction of MG53.

## 1 INTRODUCTION

Bone marrow stem cells (BMSCs) are important sources for cell‐based therapy that remains a viable and attractive option for tissue repair and regeneration.^1^, ^2^, ^3^, ^4^, ^5^ However, one of the major challenges for cell‐based therapy with stem cells is the poor in vivo survival after delivery into target areas.^4^, ^5^ It has been shown that the number of mesenchymal stem cells (MSCs) in the cremaster decreased to 14% of the initial number 3 days after bolus injection into the ipsilateral common iliac artery in rats.^6^ MSCs were initially accumulated in the lungs and did not reach the target sites after intravenous infusion, and many cells disappeared 2 hours after delivery in mice.^7^ When the stem cells were injected into the left ventricular myocardium of mice, less than 1% of the delivered cells survived in the target areas 4 days after injection.^4^

The mechanisms for the poor survival of transplanted BMSCs have not been fully understood and are very likely multifactorial including inflammation, oxidative stress, mechanical stress and hypoxia. Oxidized low‐density lipoproteins (ox‐LDLs) are naturally present in serum and an important source for reactive oxygen species (ROS) and oxidative stress.^8^, ^9^ Ox‐LDL has been shown to inhibit proliferation and endothelial differentiation of BMSCs, and induce apoptosis of BMSCs with both ROS‐dependent and ROS‐independent mechanisms.^10^, ^11^ Our previous study showed that ox‐LDL impaired the survival of BMSCs in vitro partially through direct cell membrane damage independent of ROS formation.^12^

MG53 (also known as TRIM72) is an important membrane‐repairing protein that is produced in striated muscle cells and present in circulation.^13^ Systemic delivery of MG53 or muscle‐specific overexpression of human MG53 gene enhanced membrane repair and improved muscle and heart functions in a hamster model of muscular dystrophies and congestive heart failure. MG53 could protect muscle cells by activating cell survival kinases, such as Akt, extracellular signal‐regulated kinases (ERK1/2) and glycogen synthase kinase‐3β, and inhibiting proapoptotic protein Bax.^14^ Extracellular MG53 protein and recombinant human MG53 protein (rhMG53) could increase membrane repair capacity in isolated muscle or non‐muscle cells in a dose‐dependent manner.^15^, ^16^ In our previous study, we observed that rhMG53 treatment protected BMSCs against ox‐LDL‐induced membrane damage and enhanced their survival. However, the protective effect of rhMG53 on BMSCs against ox‐LDL was limited with undefined mechanisms.

It is known that ischaemia/reperfusion or hypoxia/oxidative stress leads to down‐regulation of MG53 in rodent cardiomyocytes.^17^ Hypercholesterolaemia could block sevoflurane‐induced cardioprotection against ischaemia‐reperfusion injury by alteration of MG53‐mediated pathway.^18^ Little is known on the metabolism of MG53 in vivo. However, it has been shown that serum MG53 levels in mice with metabolic syndrome induced by a 6‐month high‐fat diet were significantly reduced.^19^ S‐nitrosylation of MG53 at C144 (cysteine 144) prevented oxidation‐induced degradation of MG53 following oxidative insult, therefore enhancing cardiomyocyte survival.^20^ In the present study, we tested the hypothesis that the limited protective effect of MG53 on BMSCs against ox‐LDL was due to ox‐LDL‐induced reduction of MG53. Both in vitro and in vivo experiments were conducted to test the hypothesis. The objectives of the present study were (a) to determine the effect of ox‐LDL on MG53 levels both in vitro and in vivo and (b) to define the role of ROS in mediating the effect of ox‐LDL‐induced reduction of MG53 by blocking ROS production with antioxidant N‐acetylcysteine (NAC).

## 2 MATERIALS AND METHODS

### 2.1 Animals

All the animal experiments were performed in accordance with the ‘Guide for the Care and Use of Laboratory Animals of the US National Institutes of Health’. The experimental protocols for the present study were reviewed and approved by the Institutional Animal Care and Use Committee of the University of Missouri School of Medicine. Male C57 BL/6 and LDL receptor deficiency (LDLR^−/−^) mice (6‐8 weeks old) were obtained from Jackson Lab.

### 2.2 Preparation of LDL and ox‐LDL

Both native LDL and ox‐LDL were prepared for the experiments as described.^10^, ^21^, ^22^ Briefly, plasma was obtained from healthy human volunteers for the preparation of native LDL using sodium bromide stepwise density gradient centrifugation. To prepare ox‐LDL, native LDL was exposed to copper sulphate (5 μmol/L) at 37°C for 3 hours, followed by the addition of EDTA (final concentration of 0.25 mmol/L) to terminate the reaction. The degree of LDL oxidation was monitored using thiobarbituric acid reactive substances (TBARS) as described.^10^, ^21^, ^22^ To ensure product quality and reproducibility, the TBARS value for ox‐LDL was maintained in the range of 40‐50 nmol malondialdehyde/mg protein. There were no detectable TBARS for native LDL.

### 2.3 Preparation of rhMG53

High‐quality (>97% purity) rhMG53 protein was prepared using E. coli fermentation as described.^16^ The efficacy of rhMG53 on membrane protection was determined as EC50 for each preparation to ensure product quality and reproducibility with our established micro‐glass bead injury assay as described.^16^, ^23^ The amount of rhMG53 protein for each experiment was determined to achieve its EC50 concentration as established by micro‐glass bead injury assay.

### 2.4 Cell culture

Rat bone marrow multipotent adult progenitor cells (MAPCs) were used as the source of BMSCs that were prepared and characterized in Dr Verfaillie's laboratory in the Stem Cell Institute at the University of Leuven, Leuven, Belgium. Rate MAPCs were stable phenotypically and were positive for Oct‐4, Rex‐1, c‐Kit and Pdgfr‐a and negative for Sca‐1, CD34, CD45, Sox‐2 and Nanog as extensively described previously.^10^, ^24^, ^25^ The cells were cultured in 60% low glucose Dulbecco's Modified Eagle's Medium (DMEM) (Gibco) and 40% MCDB‐201 at pH 7.2, supplemented with 1000 units/mL leukaemia inhibitory factor (LIF; Esgro Chemicon), 2% foetal bovine serum (FBS; HyClone), 1× insulin‐transferrin‐selenium (ITS), 1× linoleic acid‐bovine serum albumin (LA‐BSA), 100 IU/mL penicillin and 100 µg/mL streptomycin, 10^−4^ mol/L L‐ascorbic acid (add 256 mg of L‐ascorbic acid to 100 mL PBS), 10 ng/mL human platelet‐derived growth factor (PDGF), 10 ng/mL mouse epidermal growth factor (EGF, Sigma), 0.05 µmol/L dexamethasone and 55 µmol/L 2‐mercaptoethanol. Culture dishes were coated with 100 ng/mL fibronectin (FN; Sigma) to accelerate cell adherence. Cells were strictly kept at a density of 100‐200 cells/cm^2^ to avoid cell‐cell contact at 37°C with humidified gas mixtures of 5% O_2_, 5% CO_2_ and 90% N_2_.

To investigate the effect of ox‐LDL on the growth and survival of MAPCs, the cells were cultured at a density of 500 cells/cm^2^ (1000 cells/well in 24‐well plate) in the presence of ox‐LDL (10 μg/mL) for 12, 24 and 48 hours, or cultured with 20 μg/mL ox‐LDL for 24 hours at a density of 1 × 10^4^ cells/cm^2^ (as the majority of cells could die out within 24 hours of exposure to ox‐LDL at this concentration). Native LDL and saturated LDL were used as the controls. To determine whether rhMG53 could protect the cells, purified rhMG53 (1 mmol/L) was mixed with the culture medium 5 minutes before exposure to ox‐LDL. Bovine serum albumin (BSA) (1 mmol/L) was used as control. To determine the involvement of ROS in the protection of rhMG53 on MAPCs, experiments were repeated when the antioxidant NAC (1 mmol/L, final concentration) was added into the culture system 1 minute before rhMG53 was mixed with the cells. The cells were counted in each group at each time‐point, and each experiment was repeated for at least three times.

### 2.5 Measurement of rhMG53 in culture system with Western blot

rhMG53 was added into culture plates with PBS at the same concentration as with cell culture (final concentration of 1 mmol/L). To investigate the effect of ox‐LDL on rhMG53, ox‐LDL (final concentration of 10 μg/mL) was mixed with rhMG53 in PBS in culture plates. To determine whether NAC could protect rhMG53 against ox‐LDL‐induced reduction, experiments were repeated when NAC (1 mmol/L) was mixed with ox‐LDL with or without rhMG53 in PBS. The preparations were in duplicate for each sample in one 96‐well cell culture plate (100 μL total volume per well) and incubated at 37°C with 5% O_2_, 5% CO_2_ and 90% N_2_ as for cell culture. To determine whether phospholipid‐containing liposome could have an important impact on MG53 level in vitro, experiments were conducted to incubate MG53 with native LDL (10 μg/mL). After 12 hours, 50 μL sample was removed from each well and mixed with 450 μL PBS in a 1.5 mL tube; then, 10 μL sample from each tube was mixed with 10 μL protein loading buffer (Bio‐Rad Laemmli sample buffer without B‐mercaptoethanol). Without heating at 95°C, the samples (a total of 10 μL for each sample) were loaded on 10% SDS‐polyacrylamides gels (Bio‐Rad) for electrophoresis and then transferred to polyvinylidene difluoride membranes (PVDF, Millipore). After blocking with milk (Bio‐Rad), the membranes were incubated overnight at 4°C with the primary antibody for MG53. After washing with TBST, the preparations were incubated with peroxidase‐conjugated secondary antibody for 1 hour. The protein bands on the membranes were visualized using the enhanced chemiluminescence reagents (Thermo Fisher Scientific Inc) and analysed with Fiji image software.

### 2.6 Measurement of MG53 in serum from mice with or without ox‐LDL treatment

Wild‐type (WT) male C57 BL/6 mice were divided into 3 groups: (a) mice with injection of Sat‐LDL once daily for 3 days via tail vein with normal drink water (control group); (b) mice with injection of ox‐LDL (25 μg per mouse once daily for 3 days) via tail vein with normal drink water; and 3) mice with injection of ox‐LDL (25 μg per mouse once daily for 3 days) via tail vein with NAC in drink water for 1 week prior to ox‐LDL injection. Blood was harvested via cardiac puncture from the mice 24 hours after last injection of ox‐LDL to determine serum MG53 level. After heating at 95°C for 5 minutes, the serum preparations [2 μL serum + 4 μL PBS + 6 μL loading buffer (Bio‐Rad, Laemmli sample buffer: B‐mercaptoethanol of 95:5)] from each group were loaded on 8.725% SDS‐polyacrylamides gels (Bio‐Rad) for electrophoresis and then transferred to polyvinylidene difluoride membranes (PVDF, Millipore). After blocking with milk (Bio‐Rad), the membranes were incubated overnight at 4°C with the primary antibody against MG53. After washing with TBST, the protein bands on the membranes were visualized using the enhanced chemiluminescence reagents (Thermo Fisher Scientific Inc) and analysed with Fiji image software.

### 2.7 Evaluation of serum MG53 clearance in mouse

Serum MG53 level is the combined outcome of its secretion, degradation and clearance from blood. To determine whether ox‐LDL could have significant impact on MG53 clearance from circulation, we generated maltose‐binding protein‐conjugated MG53 (MBP‐MG53) specifically to differentiate endogenous MG53 from the injected MG53. The MBP‐MG53 protein was injected into WT C57BL/6 mice via tail vein (single bolus, 25 μg per mouse) with and without ox‐LDL treatment (25 μg per mouse once daily for 3 days) via tail vein injection. Sat‐LDL was used as control. A series of small blood samples were collected via tail vein at different time (0, 10, 30, 60 and 120 minutes and 4, 6 and 8 hours) after injection to measure serum MBP‐MG53 level using Western blot to determine the half‐life of MBP‐MG53 as described above.

### 2.8 Western blot analysis for Akt and STAT3 expressions

Rat MAPCs were homogenized and centrifuged at 14 500 *g* for 10 minutes, and the supernatant was collected. Protein concentration was determined using the bicinchoninic acid assay. The protein samples (20 μg) were loaded into 8% acrylamide SDS gel. The proteins were transferred to 0.45‐mm PVDF membranes (Millipore) and then incubated with 5% non‐fat milk. After 1 hour, the preparations were incubated with primary antibodies for total Akt (1:3000, Cell Signaling), phospho‐Akt (Ser473) (1:2000, Cell Signaling), total STAT3 (1:3000, Cell Signaling), phospho‐STAT3 (Tyr705) (1:1000, Cell Signaling) and GAPDH (1:10000, Sant Cruz). Subsequently, the preparations were incubated with HRP‐conjugated secondary antibodies. The blots were then incubated with chemiluminescent substrate (Thermo Scientific), and the bands were detected with X‐ray film exposure and analysed using ImageJ software.

### 2.9 FM1‐43 dye entry detection

Rat MAPCs were seeded in 35 mm glass bottom dishes at a density of 1 × 10^4^ cells/cm^2^ and cultured overnight. The cells were then treated with ox‐LDL (10 μg/mL) with or without rhMG53 (at EC_50_) in the presence or absence of NAC (1 mmol/L) for 24 hours with PBS and BSA as controls. After rinsing with Tyrode's solution (137 mmol/L NaCl, 2.7 mmol/L KCl, 1 mmol/L MgCl_2_, 1.8 mmol/L CaCl_2_, 0.2 mmol/L Na_2_HPO_4_, 12 mmol/L NaHCO_3_ and 5.5 mmol/L D‐glucose), the cells were mixed with FM1‐43 dye. The water‐soluble FM1‐43 is non‐toxic to cells and non‐fluorescent in aqueous medium. It becomes intensely fluorescent when it enters injured cells and binds to cellular lipids.^14^, ^16^, ^23^, ^26^ Entry of the FM1‐43 dye into the cells was quantitatively monitored continuously with fluorescence confocal microscope (Zeiss LSM780) immediately after mixing MAPCs with the dye. Live cell images were obtained consecutively at an interval of 4.1 s/frame for a total of 100 frames and quantitatively analysed with Fiji software as described.^27^

### 2.10 Cell proliferation assay

Rat MAPCs were seeded on a 96‐well plate at a density of 1000 cells/well in the presence of ox‐LDL (5‐10 μg/mL) for 24 hours. Each treatment was in triplicate, and three independent experiments were performed. To evaluate the effect of NAC (1 mmol/L) and/or rhMG53 (50 μg/mL) on cell proliferation and survival, NAC and/or rhMG53 were added to the culture medium 30 minutes before exposure to ox‐LDL. After 24 hours of incubation, the cells were prepared for proliferation assay using BrdU Proliferation Assay Kit (Calbiochem) as per manufacturer's instruction.

### 2.11 Cell apoptosis assay

Rat MAPCs were plated on 6‐well plates with a density of 2000 cells/cm^2^ for apoptosis assay. After 24 hours of culture, the cells were treated with ox‐LDL (5‐10 μmol/L) for an additional 24 hours with or without NAC (1 mmol/L) and/or MG53 (50 μg/mL). Each treatment was in triplicate, and three independent experiments were performed. The cells were then prepared for apoptosis assay using FITC Annexin V Apoptosis Detection Kit (Calbiochem) as per manufacturer's protocol. The proportion of apoptotic cells was expressed as a percentage of total cell number acquired (excluding debris) and analysed using BD FACS Diva and Flow Jo software.

### 2.12 Cell cycle assay

Rat MAPCs were plated on 6‐well plates with a density of 2000 cells/cm^2^ for cell cycle analysis. After 24 hours of culture, the cells were treated with ox‐LDL (5‐10 μmol/L) for an additional 24 hours with or without NAC (1 mmol/L) and/or MG53 (50 μg/mL). Each treatment was in triplicate, and three independent experiments were performed. The cells were then prepared for cell cycle analysis using BrdU/7‐AAD kit (Biolegend) according to manufacturer's protocol.

### 2.13 Statistical analysis

The data from all experiments were presented as mean ± SD (standard deviation). Statistical analyses were performed with unpaired Student's *t* test (two‐sided) for two group of data or one‐way ANOVA (analysis of variance) (Sigma Stat 2.03; Aspire Software International) followed by *post hoc* conservative Tukey's test for three or more groups of data with multiple comparisons to minimize the type I error as appropriate. The difference was considered statistically significant when a two‐tailed *P* value was equal to or less than .05.

## 3 RESULTS

### 3.1 NAC significantly enhanced the protective effect of rhMG53 on MAPCs against ox‐LDL‐induced inhibition

**Figure 1:**
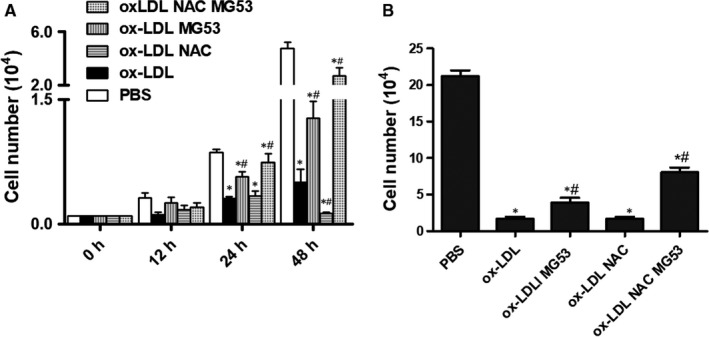
Interaction between N‐acetylcysteine (NAC) and rhMG53 on MAPCs in the presence of ox‐LDL. Normal growth of MAPCs was observed under the standard culture conditions. The number of MAPCs in the culture system was significantly decreased in the presence of ox‐LDL. When ox‐LDL concentration was at 10 μg/mL, NAC treatment (1 mmol/L) did not prevent ox‐LDL‐induced reduction of the number of MAPCs. Treatment with rhMG53 (at EC50 level) significantly improved the cell number in the presence of 10 μg/mL ox‐LDL, but not completely restored the cell number to the control level. Treatment with rhMG53 in combination with NAC almost completely restored the cell number to the control level (BSA). NAC treatment did not prevent ox‐LDL‐induced reduction in cell number at 24 h, and yet, NAC further decreased ox‐LDL‐induced reduction in cell number at 48 h (A). When ox‐LDL concentration was increased to 20 μg/mL, the number of MAPCs was only about 1/10 of that cultured with PBS or Sat‐LDL (control) after 24 h. Treatment with NAC did not improve ox‐LDL‐induced reduction of cell number at 24 h. However, compared with BSA control, treatment with rhMG53 doubled the cell number and further increased the cell number when both NAC and MG53 were present (B). PBS: cells treated with PBS or sat‐LDL (control); ox‐LDL: cells treated with ox‐LDL; ox‐LDL + NAC: cells treated with ox‐LDL and NAC; ox‐LDL + MG53: cells treated with ox‐LDL and MG53; and ox‐LDL + NAC+MG53: cells treated with ox‐LDL and NAC as well as MG53. Data were presented as means ± SEM. *P < .01 as compared with control, ^#^ *P* < .01 as compared with ox‐LDL (n = 3 independent experiments)

Healthy growth of MAPCs was observed under the standard conditions. In the presence of ox‐LDL (from 1 to 5 μg/mL), the number of MAPCs in the culture system was significantly decreased as expected. Treatment with NAC (1 mmol/L) effectively prevented ox‐LDL‐induced reduction of the cell number (data not shown). However, when ox‐LDL concentration was increased to 10 μg/mL, NAC treatment did not prevent the reduction of cell number that was consistent with our previous observations.^10^, ^12^ As expected, treatment with rhMG53 (at EC50 level) significantly improved the cell number in the presence of 10 μg/mL ox‐LDL, but not completely restored the cell number to the control level. Interestingly, rhMG53 treatment in combination with NAC almost completely restored the number to the control level (BSA). NAC treatment had no improvement in ox‐LDL‐induced reduction in cell number at 24 hours, and yet NAC further decreased ox‐LDL‐induced reduction in cell number at 48 hours (Figure 1A). When ox‐LDL concentration was increased to 20 μg/mL, the number of MAPCs was only about 1/10 of that cultured with PBS or Sat‐LDL (control) after 24 hours. Treatment with NAC could not improve ox‐LDL‐induced reduction of cell number at 24 hours. However, compared with the BSA control, treatment with rhMG53 doubled the cell number and further increased the cell number when both NAC and MG53 were present (Figure 1B).

### 3.2 NAC effectively prevented ox‐LDL‐induced reduction of MG53 protein in vitro and in vivo

**Figure 2:**
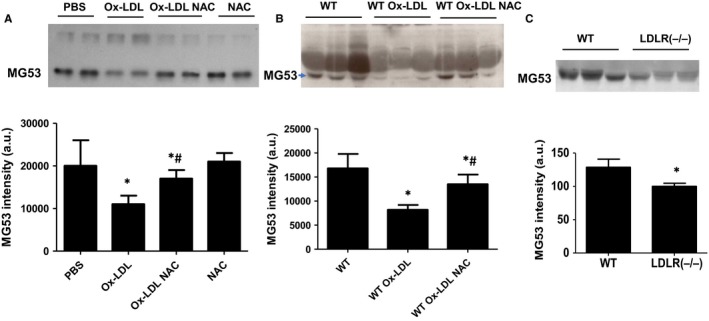
Effect of N‐acetylcysteine (NAC) on the level of rhMG53 protein in vitro and in vivo with and without ox‐LDL. MG53 level was determined in culture system in the presence of ox‐LDL with and without NAC. After 12 h of incubation in the culture environment for MAPCs, a detectable amount of rhMG53 protein was present in the culture media. Ox‐LDL (10 μg/mL) significantly reduced the level of rhMG53 protein in the culture system with or without MAPCs. The presence of NAC (1 mmol/L) effectively prevented ox‐LDL‐induced reduction of rhMG53 protein level in the culture system (A). Serum levels of MG53 protein in wild‐type (WT) mice were determined with Western blotting after three consecutive days of tail vein injection of ox‐LDL with or without NAC treatment. Serum MG53 was readily detectable in WT mice and was significantly decreased in mice treated with ox‐LDL. Pre‐treatment of the mice with NAC effectively prevented ox‐LDL‐induced decrease in serum MG53 level (as pointed with the arrow, B). Of note, serum MG53 level in male hyperlipidemic LDLR(‐/‐) mice after 8 wk of high‐fat diet was significantly lower than the serum MG53 level in male age‐matched WT mice (C). PBS: cells treated with PBS or sat‐LDL (control); ox‐LDL: cells treated with ox‐LDL; ox‐LDL + NAC: cells treated with ox‐LDL and NAC; NAC: cells treated with NAC (control); WT: control mice treated with PBS or sat‐LDL; WT + ox‐LDL: mice treated with ox‐LDL; and WT + ox‐LDL + NAC: mice treated with ox‐LDL and NAC. LDLR(‐/‐): hyperlipidemic LDL receptor knockout mice with 8 wk of high‐fat diet. Data were presented as means ± SEM. *P < .01 as compared with control, ^#^ *P* < .01 as compared with ox‐LDL (n = 3 independent experiments)

It is known that the protective effect of MG53 on cell membrane is dose‐dependent.^16^ To test the hypothesis that NAC enhanced the protective effect of MG53 on MAPCs against ox‐LDL through the prevention of ox‐LDL‐induced reduction of MG53, MG53 level was determined in the culture system in the presence of ox‐LDL or native LDL when ROS production was blocked with NAC. As expected, after 12 hours of incubation in the MAPCs culture environment, a detectable amount of MG53 protein was present in the media. Ox‐LDL (10 μg/mL) significantly reduced MG53 level in the culture system with or without MAPCs (Figure 2A), while no reduction of MG53 level was observed with native LDL (Figure S1). The presence of NAC (1 mmol/L) effectively prevented ox‐LDL‐induced reduction of MG53 protein in the culture system (Figure 2A).

To determine whether ox‐LDL could impair MG53 level in vivo, serum MG53 levels in WT mice were determined with Western blotting after three consecutive days of tail vein injection of ox‐LDL with or without NAC treatment. Serum MG53 was readily detectable in WT mice and was significantly decreased in mice treated with ox‐LDL (Figure 2B). Pre‐treatment of the mice with NAC effectively prevented ox‐LDL‐induced decrease in serum MG53 level. Of note, serum MG53 level in male hyperlipidemic LDLR (−/−) mice after 8 weeks of high‐fat diet was significantly lower than the serum MG53 level in male age‐matched WT mice (Figure 2C).

### 3.3 Ox‐LDL had no effect on MG53 clearance in vivo

**Figure 3:**
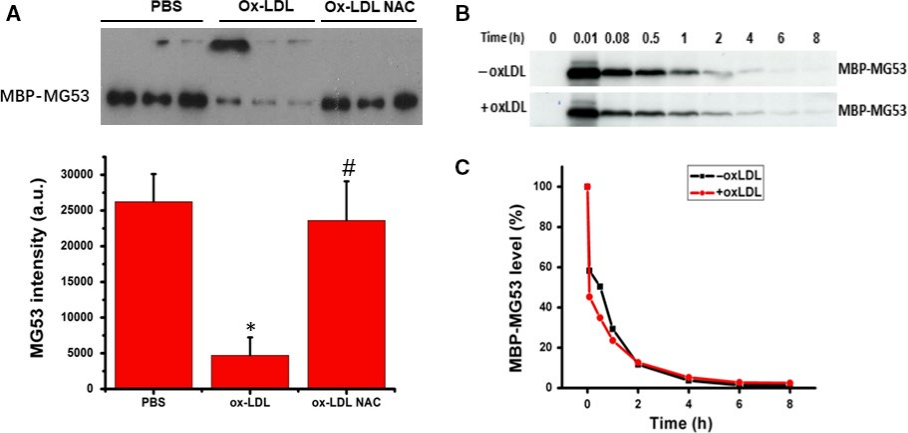
Effect of ox‐LDL on MG53 clearance in vivo. MBP‐MG53 was generated to determine MG53 clearance in vivo. Prior to injection into mice, in vitro experiments showed that ox‐LDL (10 μg/mL) significantly decreased the concentration of MBP‐MG53 after 12 h of incubation, and NAC treatment completely prevented ox‐LDL‐induced reduction of MBP‐MG53 protein (A). After injection through tail vein, serum concentration of MBP‐MG53 was decreased rapidly over time. At 30 min after injection, less than half of the injected MG53 protein was present in the circulation. After 2 h, only 10% of the injected MG53 protein remained in the blood. By 6 h, the injected protein was completely removed from the blood. Treating mice with ox‐LDL (once a day for 3 d via tail vein) had no significant effect on MG53 clearance from circulation (B). MBP‐MG53: maltose‐binding protein‐conjugated MG53; PBS: cells treated with PBS or sat‐LDL (control); ox‐LDL: cells treated with ox‐LDL; ox‐LDL + NAC: cells treated with ox‐LDL and NAC; ox‐LDL: wild‐type mice treated with ox‐LDL; and ‐ox‐LDL: control mice without ox‐LDL treatment. Data were presented as means ± SD. *P < .01 as compared with control, ^#^ *P* < .01 as compared with ox‐LDL (n = 3 independent experiments)

Next, we determined whether ox‐LDL could have a significant impact on MG53 clearance in vivo that might contribute to the reduction of serum MG53 level. We generated MBP‐MG53 to distinguish endogenous MG53 from the injected MG53. Prior to injection into mice, in vitro experiments showed that ox‐LDL (10 μg/mL) significantly decreased the concentration of MBP‐MG53 after 12 hours of incubation, and NAC treatment completely prevented ox‐LDL‐induced reduction of MBP‐MG53 protein (Figure 3A). After injection through tail vein, serum concentration of MBP‐MG53 was decreased rapidly over time. At 30 minutes after injection, less than half of the injected MG53 protein was present in circulation. After 2 hours, only 10% of the injected MG53 protein remained in the blood. By 6 hours, the injected protein was completely removed from the blood. Treating the mice with ox‐LDL (once a day for 3 days via tail vein) had no significant effect on MG53 clearance from circulation (Figure 3B).

### 3.4 NAC treatment did not prevent ox‐LDL‐induced membrane damage of MAPCs in vitro

**Figure 4:**
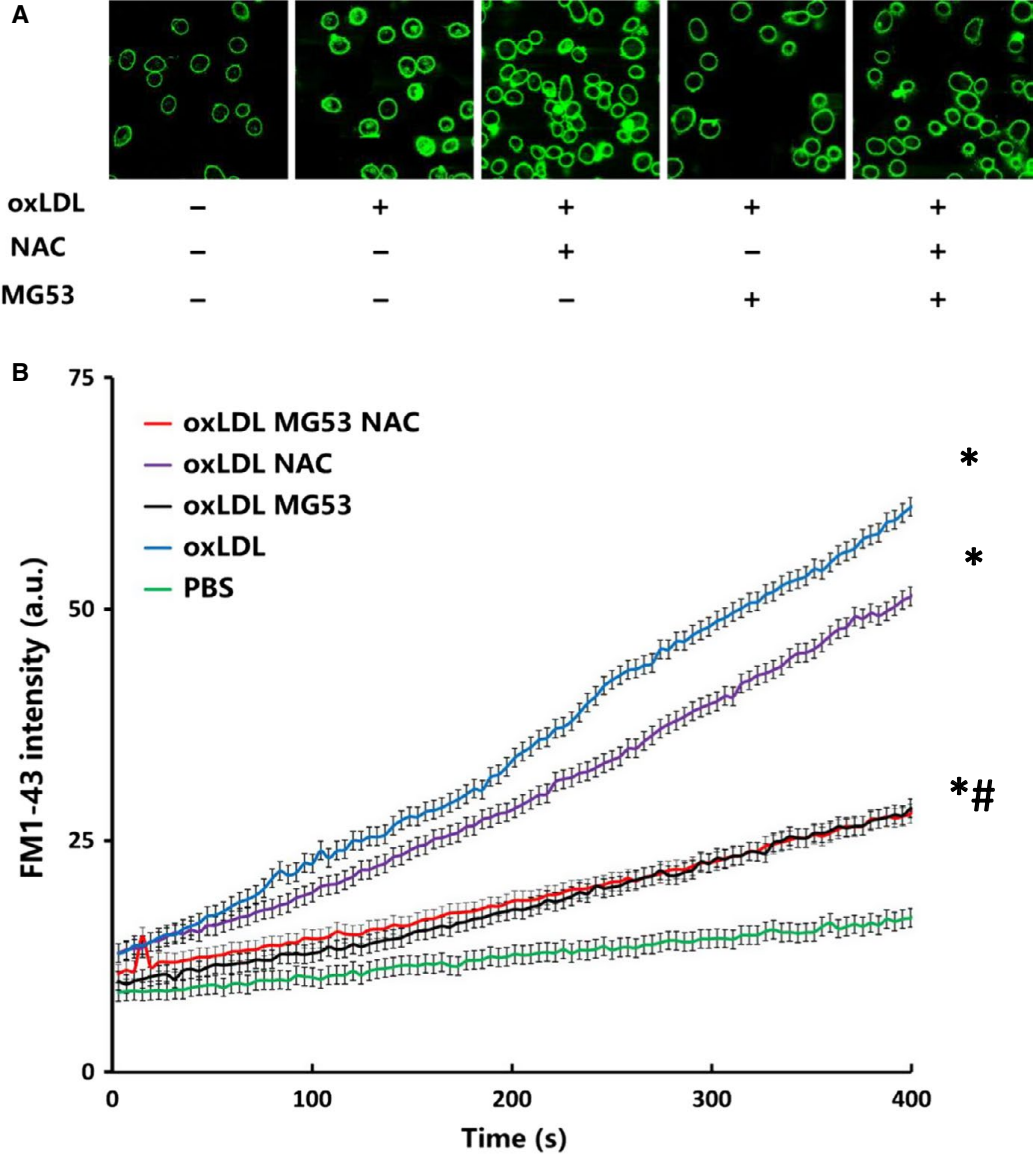
Effect of NAC on ox‐LDL‐induced membrane damage of bone marrow stem cells in vitro. Membrane integrity was evaluated by monitoring the entry of fluorescent FM1‐43 dye into the cells in the presence of ox‐LDL (10 μg/mL) with or without NAC treatment. A significant amount of FM1‐43 dye was detected 6 hours after incubation with ox‐LDL using confocal microscope. There was no significant difference in FM1‐43 dye entry into the cells when exposed to ox‐LDL with or without NAC treatment (A). Quantitative and dynamic analysis with a quantitative live cell imaging assay showed that exposure to ox‐LDL (10 μg/mL) dramatically increased FM1‐43 dye accumulation inside the cells that was not significantly changed with NAC treatment. The dynamic of FM1‐43 dye entry was analysed by imageJ (more than 200 cells were analysed for each condition and each experiment) (B). On the other hand, treatment with rhMG53 (either alone or with NAC) significantly reduced FM1‐43 dye entry into and accumulation inside the cells (A and B). PBS: cells treated with PBS or sat‐LDL (control); ox‐LDL: cells treated with ox‐LDL; ox‐LDL + NAC: cells treated with ox‐LDL and NAC; ox‐LDL + MG53: cells treated with ox‐LDL and MG53; and ox‐LDL + NAC + MG53: cells treated with ox‐LDL and NAC as well as MG53. Data were presented as means ± SEM. *P < .01 as compared with control, ^#^ *P* < .01 as compared with ox‐LDL (n = 3‐4 independent experiments, scale bar: 10 μm)

Membrane integrity plays a critical role in cell survival. To determine whether NAC could have protective effect on MAPCs against ox‐LDL‐induced membrane damage, membrane integrity was evaluated by monitoring the entry of fluorescent FM1‐43 dye into the cells in the presence of ox‐LDL (10 μg/mL) with or without NAC treatment. As expected, a significant amount of FM1‐43 dye was detected 6 hours after incubation with ox‐LDL using confocal microscope. There was no significant difference in FM1‐43 dye entry into the cells when exposed to ox‐LDL with or without NAC treatment (Figure 4A). Quantitative and dynamic analysis with a quantitative live cell imaging assay confirmed that exposure to ox‐LDL dramatically increased FM1‐43 dye accumulation inside the cells that was not significantly changed with NAC treatment (Figure 4B). On the other hand, treatment with rhMG53 significantly reduced FM1‐43 dye entry into and accumulation inside the cells (Figure 4A,B).

### 3.5 NAC treatment prevented ox‐LDL‐induced inhibition of Akt phosphorylation without enhancing the survival of MAPCs against ox‐LDL in vitro

**Figure 5:**
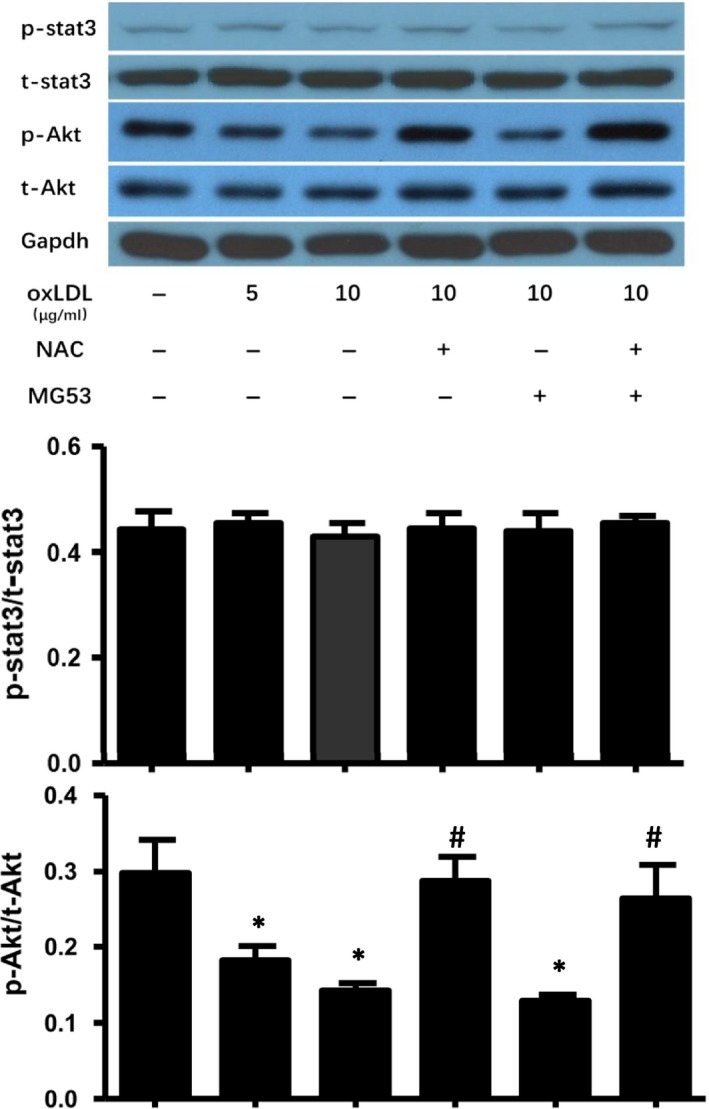
Effect of NAC and MG53 treatment on Akt phosphorylation in bone marrow stem cells in the presence of ox‐LDL in vitro. Intracellular levels of total and phosphorylated Akt and STAT3 were evaluated in MAPCs after exposure to ox‐LDL with and without NAC and/ or MG53 treatment. Western blotting analysis showed that ox‐LDL selectively decreased Akt phosphorylation without change in total Akt and total or phosphorylated STAT3 in MAPCs. NAC treatment completely prevented ox‐LDL‐induced inhibition of Akt phosphorylation in MAPCs. On the other hand, MG53 treatment did not change the levels of Akt or STAT3 expression in MAPCs exposed to ox‐LDL. PBS: cells treated with PBS or sat‐LDL (control); ox‐LDL: cells treated with ox‐LDL; ox‐LDL + NAC: cells treated with ox‐LDL and NAC; ox‐LDL + MG53: cells treated with ox‐LDL and MG53; and ox‐LDL + NAC+MG53: cells treated with ox‐LDL and NAC as well as MG53. Data were presented as means ± SEM. *P < .01 as compared with control, ^#^ *P* < .01 as compared with ox‐LDL (n = 3 independent experiments)

Akt‐ and STAT3‐mediated signalling is important to cell survival and proliferation. To determine the potential role of Akt and STAT3 signalling in mediating the effect of ox‐LDL on MAPCs, the levels of total and phosphorylated Akt and STAT3 were evaluated in the cells exposed to ox‐LDL with and without NAC treatment. As shown in Figure 5, ox‐LDL selectively decreased Akt phosphorylation without change in total Akt and total or phosphorylated STAT3 in MAPCs. NAC treatment completely prevented ox‐LDL‐induced inhibition of Akt phosphorylation in MAPCs. However, NAC treatment had no protective effect on ox‐LDL‐induced membrane damage or cell survival (Figures 1 and 4). On the other hand, MG53 significantly decreased ox‐LDL‐induced membrane damage with decreased FM1‐43 dye entry and accumulation in MAPCs (Figure 4) and, yet, did not change the levels of Akt or STAT3 expression in MAPCs (Figure 5). Combined treatment with NAC and MG53 had almost identical effect as MG53 treatment alone on FM1‐43 dye entry and accumulation in MAPCs (Figure 4), and Akt or STAT3 expression in MAPCs (Figure 5).

### 3.6 Effects of NAC on cell proliferation, apoptosis and cell cycle in the presence of ox‐LDL

**Figure 6:**
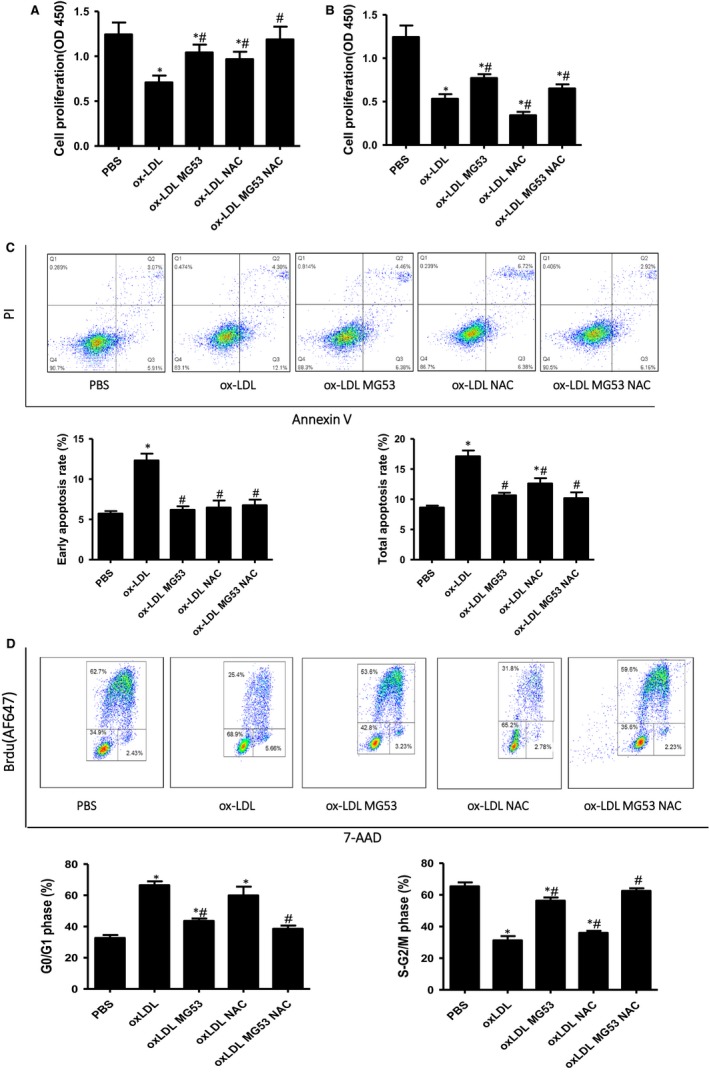
Effects of NAC on cell proliferation, apoptosis and cell cycle in the presence of ox‐LDL. MAPCs were incubated with ox‐LDL for 24 hours in the presence of MG53 with or without NAC. Ox‐LDL significantly inhibited the proliferation of MAPCs (A and B), induced apoptosis of MAPCs (C) and arrested the cell cycle at G0/G1 phase (D). Treatment with MG53 partially reversed ox‐LDL‐induced inhibition of cell proliferation (A and B), effectively prevented ox‐LDL‐induced apoptosis (C) and largely reversed ox‐LDL‐induced cell cycle arrest (D). NAC treatment significantly prevented ox‐LDL‐induced inhibition of cell proliferation and blocked ox‐LDL‐induced early apoptosis when ox‐LDL concentration was at 5 μg/mL. However, when ox‐LDL concentration was increased to 10 μg/mL, NAC treatment further increased ox‐LDL‐induced inhibition of cell proliferation (A) while having no effect on ox‐LDL‐induced cell cycle arrest (D). No significant differences in proliferation, apoptosis and cell cycle of MAPCs were observed when the cells were treated with MG53 alone or with combination of NAC and MG53 in the presence of ox‐LDL at 10 μg/mL (A‐D). PBS: cells treated with PBS or sat‐LDL (control); ox‐LDL: cells treated with ox‐LDL; ox‐LDL + NAC: cells treated with ox‐LDL and NAC; ox‐LDL + MG53: cells treated with ox‐LDL and MG53; and ox‐LDL + NAC+MG53: cells treated with ox‐LDL and NAC as well as MG53. Data were presented as means ± SEM. *P < .01 as compared with control, ^#^ *P* < .01 as compared with ox‐LDL (n = 3‐4 independent experiments)

As expected, ox‐LDL significantly inhibited the proliferation of MAPCs, induced their apoptosis and arrested the cell cycle at G0/G1 phase (Figure 6A‐D). Treatment with MG53 partially prevented ox‐LDL‐induced inhibition of cell proliferation (Figure 6A,B), effectively prevented ox‐LDL‐induced apoptosis (Figure 6C) and largely reversed ox‐LDL‐induced cell cycle arrest (Figure 6D). NAC treatment significantly prevented ox‐LDL‐induced inhibition of cell proliferation and blocked ox‐LDL‐induced early apoptosis when ox‐LDL concentration was at 5 μg/mL. However, when ox‐LDL concentration was increased to 10 μg/mL, NAC treatment further increased ox‐LDL‐induced inhibition of cell proliferation (Figure 6A) while having no effect on ox‐LDL‐induced cell cycle arrest (Figure 6D). No significant differences in proliferation, apoptosis and cell cycle of MAPCs were observed when the cells were treated with MG53 alone or with combination of NAC and MG53 in the presence of ox‐LDL at 10 μg/mL (Figure 6A‐D).

## 4 DISCUSSION

In the present study, rat MAPCs were used as the source of BMSCs as BMSCs are a mixture of heterogeneous cells with very different phenotypes and different capability of differentiating into multiple cell lineages.^28^ MAPCs were isolated from bone marrow, clonally purified and well‐characterized multipotent cells with differentiation potential into a variety of cell lineages including endothelial cells, smooth muscle cells, neurons, hepatocytes and cardiac myocytes.^24^, ^25^, ^29^, ^30^ MAPCs promote angiogenesis and improve cardiac and limb function when injected into the peri‐infarct areas in ischaemic mice.^31^, ^32^, ^33^, ^34^, ^35^, ^36^, ^37^, ^38^, ^39^, ^40^ These cells have extensive replication potential, are commercially available at clinical grade (Athersys Inc, Cleveland, Ohio) and are non‐immunogenic for alloreactive cytotoxic T lymphocyte induction, thus without potential adverse immune reactions.^41^, ^42^ MAPCs have been approved for clinical studies in human patients with multiple disease indications including neurological, cardiovascular and inflammatory and immune diseases in the United States and Europe.^43^, ^44^ Clinical‐grade human MAPCs are safe in human patients and effective on controlling human autoimmune disease and allograft rejection.^39^, ^40^, ^43^, ^44^ In addition, rat MAPCs are very stable phenotypically. Thus, using rat MAPCs in the present study has unique advantage with significant translational and clinical values.

We demonstrated that ox‐LDL at the concentrations that were compatible with serum ox‐LDL levels or less than that in patients with stable coronary artery diseases 12, 45, 46, 47 induced significant membrane damage and substantially impaired the survival of MAPCs, and selectively decreased Akt phosphorylation without change in the levels of total Akt or total and phosphorylated STAT3 in MAPCs. The antioxidant NAC effectively prevented ox‐LDL‐induced reduction of Akt phosphorylation in MAPCs, but had no protective effect on MAPCs against ox‐LDL‐induced membrane damage and impaired survival. However, the membrane repair protein MG53 had no effect on Akt phosphorylation in MAPCs, yet effectively prevented ox‐LDL‐induced membrane damage and partially improved the survival, proliferation and apoptosis of MAPCs in vitro. We also observed that ox‐LDL significantly decreased MG53 level in vitro, and in vivo without changing in vivo MG53 clearance from circulation. NAC treatment effectively prevented ox‐LDL‐induced reduction of MG53 both in vitro and in vivo. Treatment with combined NAC and MG53 significantly improved the survival of MAPCs against ox‐LDL. These data suggested that NAC enhanced the protective effect of MG53 on MAPCs against ox‐LDL through the prevention of ox‐LDL‐induced reduction of MG53 in vitro.

Membrane integrity is important for normal cell function especially cell survival. Disruption of membrane integrity or impaired membrane repair could result in pathological changes including cell death in a variety of organ systems and cells.^48^, ^49^ Recent studies have identified some proteins that are critically involved in membrane repair and maintaining the membrane integrity for cell survival and optimal cell function.^14^, ^50^, ^51^, ^52^, ^53^ MG53 is a muscle‐specific tripartite motif (TRIM) family protein with 477 amino acids and is an essential component of the acute membrane repair machinery.^52^ MG53 ablation resulted in defective membrane repair and progressive skeletal myopathy.^14^, ^54^, ^55^ MG53 knockout (MG53^−/−^) mice were susceptible to cardiac ischaemia/reperfusion (I/R) injury, whereas MG53 overexpression significantly protected cardiomyocytes from I/R damage and improved cardiac function in animal models.^17^, ^18^, ^50^, ^56^

MG53 can be secreted into circulation and mediates the protective function of MG53 for tissues and organ systems away from striated muscles.^13^ Thus, the level of circulating MG53 could be critical for tissue repair and/or regeneration and could be determined by the combined outcome of MG53 expression and secretion, degradation and clearance from circulation. However, the regulatory mechanism for serum MG53 concentration has not been well defined. It has been shown that mice with metabolic syndrome induced by 6‐month high‐fat diet (HFD) feeding exhibited a significant increase in serum lipid levels and a significant reduction in circulating MG53 level without change in MG53 expression within skeletal and cardiac muscles.^19^ In the present study, we also observed that serum MG53 level was significantly decreased in hyperlipidemic LDLR^−/−^ mice (Figure 2C). One of the important components in blood in hyperlipidemic state is ox‐LDL which is considered as a critical contributor to the development of cardiovascular diseases associated with hyperlipidemia (like atherosclerosis).^57^ In the present study, we demonstrated that treatment of mice with ox‐LDL significantly decreased serum MG53 level without change in MG53 clearance from circulation (Figure 2B and Figure 3B). In vitro experiments showed that ox‐LDL substantially decreased the level of MG53 (Figure 2A). These data suggested that hyperlipidemia and ox‐LDL might be an important determinant for serum MG53 level very likely through enhanced degradation.

MG53 protein has a cysteine residue at position 144 (C144) 20 that can be irreversibly oxidized by anoxia or hydrogen peroxide (H_2_O_2_) and associated with decreased cell survival.^20^ On the other hand, increased S‐nitrosylation (SNO) at C144 of MG53 protein would prevent the unfavourable oxidative modification of C144 by H_2_O_2_, and associated with reduced infarct size, and improved myocardial contraction.^20^, ^58^, ^59^ It is known that excessive amount of ROS is produced in hyperlipidemic states and associated with increased oxidative stress.^60^ A significant amount of ROS could be generated from ox‐LDL both in vitro and in vivo that could be effectively inhibited with NAC.^12^, ^60^ The data from the present study showed that ox‐LDL treatment significantly decreased MG53 levels both in vitro and in vivo that was effectively prevented with antioxidant NAC (Figure 2). It is possible that ox‐LDL could interact with MG53, leading to irreversible oxidative modification of MG53 and degradation. Further studies are needed to determine whether ox‐LDL could oxidize the C144 residue to form a disulphide bond and MG53 dimerization, leading to the interruption of normal structure and function of MG53. MG53 is also known to have phospholipid‐binding activity including binding to phosphatidylserine or phospholipid‐containing liposome.^23^, ^61^, ^62^ It could be possible that ox‐LDL‐induced reduction of MG53 might result from the binding of MG53 to phospholipids because ox‐LDL is composed of phospholipids. However, when MG53 was incubated with native LDL, no reduction of MG53 level was observed (Figure S1). Degradation or clearance of MG53 in circulation was not significantly affected with ox‐LDL treatment in mice (Figure 3B). These data suggested that binding to phospholipids might not be a dominant mechanism for decreased MG53 levels. However, further studies are needed to define the interactions between MG53 and phospholipids. Another potential mechanism for decreased MG53 level by ox‐LDL could be ox‐LDL‐induced reduction of MG53 secretion into the circulation that also requires further evaluation.

One of the major challenges to cell therapy with stem cells including BMSCs is the poor in vivo survival after delivery into target tissues. However, the factors that are involved in the in vivo survival of delivered stem cells have not been well defined. Our previous study showed that ox‐LDL significantly impaired the survival of MAPCs in vitro partially through direct membrane damage independent of ROS production when ox‐LDL was at 10 μg/mL or higher as NAC treatment completely blocked ROS production from ox‐LDL and yet failed to protect ox‐LDL‐induced membrane damage in MAPCs. Treatment with rhMG53 effectively protected MAPCs against ox‐LDL‐induced membrane damage, but only partially enhanced their survival.^12^ In the present study, we observed that ox‐LDL significantly decreased MG53 concentration both in vitro and in vivo that was largely (although not completely) prevented with NAC treatment, suggesting that ox‐LDL induced oxidative degradation of MG53. Combined treatment with NAC and MG53 also effectively attenuated ox‐LDL‐induced membrane damage and substantially enhanced the protective effect of MG53 on the survival of MAPCs with improved proliferation and reduced apoptosis against ox‐LDL. Of note, NAC treatment alone effectively prevented ox‐LDL‐induced reduction of intracellular Akt phosphorylation in MAPCs and was unable to protect the cells against ox‐LDL‐induced membrane damage or improve their survival against ox‐LDL, suggesting that ox‐LDL‐induced membrane damage was independent from intracellular Akt‐medicated mechanism. These data suggested that the synergistic effect between NAC and MG53 on enhanced survival of MAPCs was due to attenuation of ox‐LDL‐induced reduction of MG53. Future studies are needed to determine whether NAC could enhance the in vivo survival of MAPCs.

In conclusion, the data from the present study suggested that ox‐LDL significantly impaired the survival of MAPCs partially through direct membrane damage independent of ROS production in vitro. NAC treatment synergistically enhanced the protective effect of MG53 on the survival of MAPCs against ox‐LDL through attenuation of ox‐LDL‐induced reduction of MG53.

## CONFLICT OF INTEREST

None.

## AUTHOR CONTRIBUTIONS

Zhenguo Liu was responsible for the perception of the idea, involved in the experiment design, data analysis and interpretation, and modification and revision of the manuscript. Xin Li and Meng Jiang did the majority of the experiments, data analysis and preparations of the figures and drafted the manuscript. Tao Tan, Chandrakala Narasimhulu, Yuan Xiao, Hong Hao, Yuqi Cui, Jia Zhang, Lingjuan Liu, Chunlin Yang, Yixi Li and Catherine Verfaillie helped the preparations of research materials, animal models and data collections. Jianjie Ma, Hong Hao, Sampath Parthasarathy and Hua Zhu were involved in data analysis and interpretation. Hua Zhu was also involved in the manuscript revision.

## Data Availability

Data are available on request from the authors.
