# Supplementary figures and images for: N‐acetylcysteine prevents oxidized low‐density lipoprotein‐induced reduction of MG53 and enhances MG53 protective effect on bone marrow stem cells
Source: J Cell Mol Med. 2019 Nov 19;24(1):886–98. doi: 10.1111/jcmm.14798 (PMC6933383; doi:10.1111/jcmm.14798)

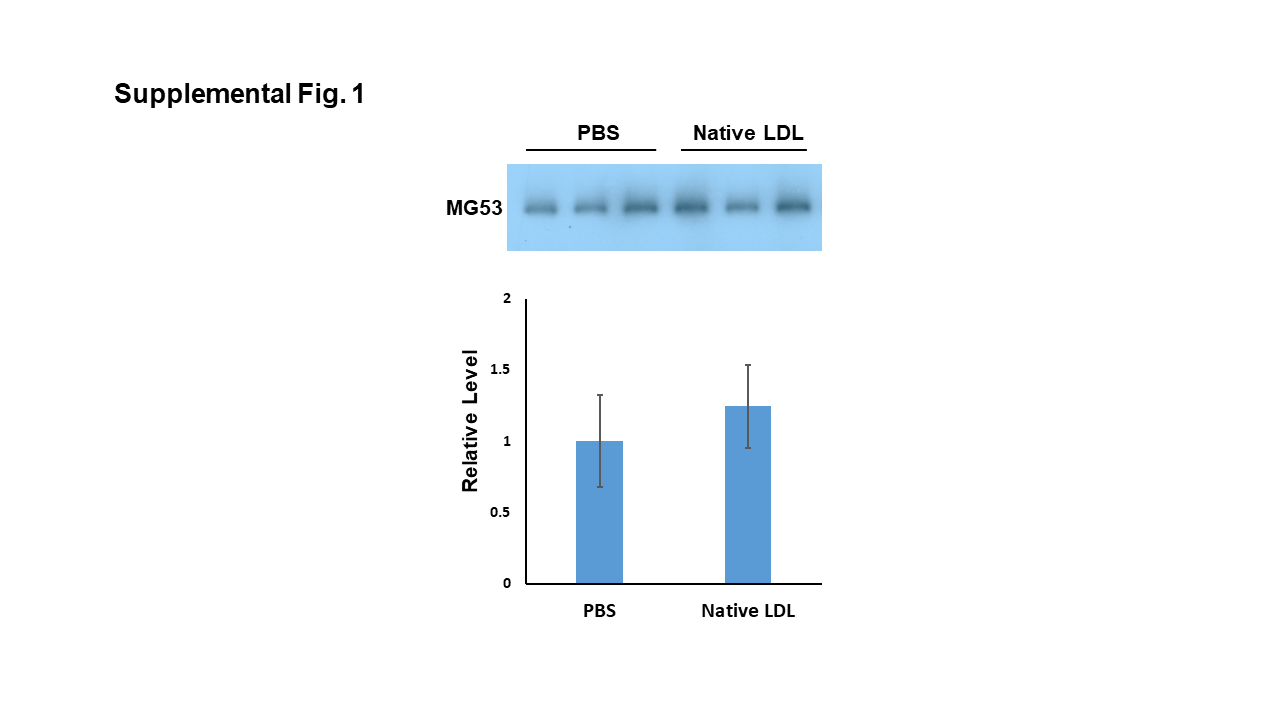

Supplement: Supplementary file 1 [file JCMM-24-886-s001.TIF]
